# Development of Variable Elastic Band with Adjustable Elasticities for Semi-Passive Exosuits

**DOI:** 10.3390/biomimetics10110734

**Published:** 2025-11-01

**Authors:** Jaewook Ryu, Gyeongmo Kim, Giuk Lee

**Affiliations:** School of Mechanical Engineering, Chung-Ang University, Seoul 06974, Republic of Korea; wodnr1958@cau.ac.kr (J.R.); woogen1206@cau.ac.kr (G.K.)

**Keywords:** variable elastic band, elasticity, force–elongation curve, semi-passive exosuit, hip-flexion assistance, Mooney–Rivlin model

## Abstract

Active exosuits provide various assistive force profiles but are limited by battery life, weight, and complex maintenance requirements. Passive exosuits, by contrast, are economical and lightweight while also offering unlimited usage times; however, due to their fixed stiffness levels, they can provide only a limited set of optimized assistive force profiles for different movements. To address these issues, this paper proposes a new variable elastic band for semi-passive exosuits. It comprises rubber bands and webbings connected in parallel, with the elongation of the rubber bands restricted according to the webbing length. By connecting these segments in series, a range of elasticities can be generated. Experimental results confirmed that the band could generate different stiffness levels, which were accurately predicted with an average coefficient of determination (R^2^) of 0.9985 and an average root mean square error of 0.8993. Additionally, based on tests involving participants wearing the device, the variable elastic band effectively modulated the assistive force profile. These findings overcome the previous limitations of passive components, opening the door to future research on enhancing the efficiency of passive systems and enabling further customization.

## 1. Introduction

Exosuits have gained significant attention due to their advantages over traditional exoskeletons. Unlike rigid exoskeletons, which attach to the body and directly drive the joints, exosuits use soft, clothing-like materials that conform to the wearer’s body and employ motors that transmit power through flexible components such as cables and webbing. They incur lower metabolic penalties due to the use of lightweight and low-inertia textile materials and ensure more natural kinematics without interrupting movement or causing joint misalignment [1,2,3,4,5]. However, active exosuits that utilize actuators have several limitations in terms of convenient usage. For instance, the battery power limits the usage duration, while the use of multiple electronic components, including actuators, batteries, circuit boards, and sensors, increases both cost and weight. Additionally, operating and maintaining active exosuits require expertise.

To overcome these limitations, passive exosuits have been developed, which enhance movement efficiency by leveraging the energy-storage and energy-release properties of elastic materials such as rubber and springs, instead of utilizing actuators [6,7,8]. Passive exosuits are lightweight, easy to wear, and inexpensive; moreover, as they do not require batteries, their usage duration is not limited. However, most passive exosuits have the drawback of a fixed assistive force profile, which can be effective for specific wearers and movements but ineffective or intrusive for others. Therefore, variability in the assistive force profile is essential for passive exosuits to accommodate various wearers and movements.

In line with this argument, numerous studies have demonstrated that the assistive effect in passive exoskeletons varies according to the force profile and that the optimal profile differs depending on the wearer and movement. It has been confirmed that metabolic cost varies with assistive stiffness for a passive ankle exoskeleton [9]. According to studies on the use of passive hip-flexion exosuits for walking [7,8] and for running [6], the band force conditions resulting in the highest metabolic reduction differed among participants. An evaluation of a passive hip-flexion exoskeleton proved that the spring stiffness that maximizes the metabolic reduction varies between walking and running [10].

In addition, human-in-the-loop (HITL) optimization studies in wearable robots have reported that not only the magnitude but also the curve shape of the assistive force profile significantly influences assistance outcomes, with each individual showing a different optimal profile [11,12,13]. Thus, if the assistive force profile in passive exosuits can be adjusted in terms of both magnitude and shape, optimal force profiles tailored to individual wearers and movements can be constructed, which would maximize efficiency.

In response to this need, several research groups have explored mechanisms that provide elastic forces of varying magnitudes and shapes. Diller et al. proposed an electroadhesive clutch-spring system that adjusts stiffness by controlling the number of stretched rubber springs connected in parallel using an electroadhesive clutch [14]. However, this device can only set stiffness levels as multiples of the rubber’s inherent elasticity, and it cannot modify the shape of the elastic force curve. Helps et al. developed an artificial muscle that can adjust stiffness based on the twist angle of a rubber cord [15]. Nevertheless, the curve shape based on the twist angle is fixed, making it impossible to adjust the elastic force profile. Furthermore, its integration into passive exosuits is challenging due to abnormal buckling behavior and increased pre-load. Bidgoly et al. developed a rotational nonlinear spring mechanism that utilizes a non-circular cam and a linear spring to implement a target torque profile [16]. Similarly, Jutte et al. designed a nonlinear leaf spring with a desired load–displacement function using a genetic algorithm and finite element analysis [17]. While these two studies can create the curve shape of a desired elastic profile, the stiffness is fixed to the designed profile, preventing immediate changes. In summary, most existing research offers systems that either allow immediate adjustment of the magnitude of elastic force or possess a fixed target curve shape. However, no system currently allows for the immediate adjustment of both the magnitude and the shape of the elastic force. Consequently, this limits the diversity of assistive force profiles that can be effectively utilized in exosuits.

Herein, we propose a variable elastic band mechanism capable of generating a wide variety of elasticities. By coupling an elastic rubber band in parallel with an inelastic webbing, the tensile length is restricted depending on the length difference between the band and the webbing. By connecting these band–webbing sections in series and adjusting the webbing length, various stiffness levels can be generated based on the different tensile lengths for each section. The system configuration, working principle, and setting the segment lengths of the variable elastic band are described in the Materials and Methods section. Experimental validation of the variable elastic band and an application test for a hip-flexion exosuit with variable elastic bands were conducted by measuring stiffness levels and assistive force profiles. Benchtop experiments confirmed that the proposed variable elastic band could accurately generate multiple stiffness levels, while preliminary walking trials demonstrated consistent modulation of assistive force profiles. These findings highlight the feasibility and effectiveness of the mechanism in both controlled and wearable conditions.

## 2. Materials and Methods

### 2.1. System Configuration

**Figure 1:**
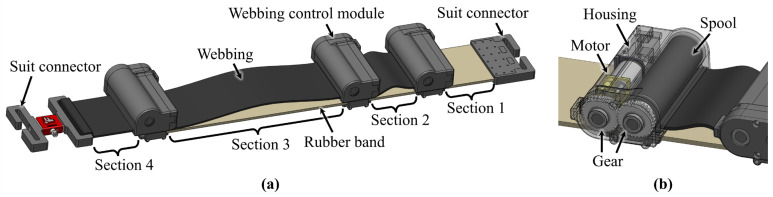
Structure of the variable elastic band. (**a**) Overview of the band components. (**b**) Internal components of the webbing control module.

As shown in Figure 1a, the variable elastic band consists of a rubber band, webbings, webbing control modules, and suit connectors. The band is divided into four elastic sections by the webbing control modules. Section 1 is left without a webbing connection to ensure that at least one section remains fully elastic, while Section 2 and Section 3 include both a rubber band and webbing. Section 4 includes solely webbing, without a rubber band, enabling adjustment of the initial length of the band.

The materials and coupling methods for the variable elastic band were selected based on the specific requirements for an exosuit. A high-tensile-strength rubber band (Super-Stretchable Natural Rubber Strip, McMaster-Carr, Elmhurst, IL, USA) was chosen considering its superior elasticity, while seat-belt webbing, known for its flexibility and strength, was used to ensure the necessary rigidity in the inelastic component. The rubber band, webbing, and webbing control modules are securely sewn together for durability and compactness. The materials for the rigid components of the variable elastic band were selected to prevent deformation while minimizing overall weight. The spools, which are subject to potential twisting, were machined using Aluminum 7075 T6, and the other components were manufactured using a 3D printer (Mark 2, Markforged, Waltham, MA, USA) with carbon fiber-reinforced Onyx.

Figure 1b shows the configuration of the webbing control module, which includes a small motor (1000:1 Micro Metal Gearmotors HPCB 6V, Pololu, Las Vegas, NV, USA), gears, a spool, and housing. One end of the webbing is connected between the module’s housing and the rubber band, while the other end is fixed to a spool in another module. The length of each webbing is precisely adjusted by rotating the spool through motor position control. The three motors used in the variable elastic band are each connected to an encoder (Magnetic Encoder Pair Kit with Top-Entry Connector for Micro Metal Gearmotors, Pololu, Las Vegas, NV, USA) and a motor driver (DRV8838 Single Brushed DC Motor Driver Carrier, Pololu, Las Vegas, NV, USA), all of which are controlled using a single main controller (Teensy 4.1, PJRC, Portland, OR, USA) via proportional–integral–derivative control. The total mass of the variable elastic band configured in this way is 270 g. The detailed composition of the variable elastic band, including electronic and mechanical subassemblies, is summarized in Table 1.

### 2.2. Working Principle

The variable elastic band stores and releases energy during walking as the suit connectors, attached to the waist and thigh parts of the hip-flexion exosuit, are stretched and relaxed, respectively. The webbing in each section restricts the elongation length of the rubber band within that section, generating a composite stiffness based on the combination of the stiffness levels of each section connected in series.

**Figure 2:**
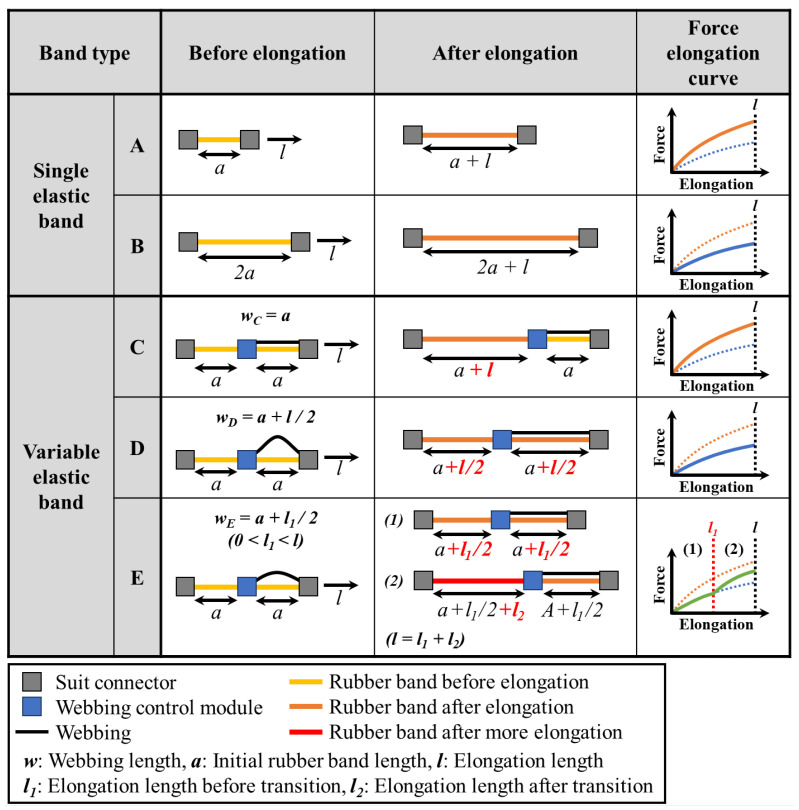
Appearance of single rubber bands and variable elastic bands with different webbing lengths before and after elongation, and their corresponding force–elongation curves.

Figure 2 illustrates how the force–elongation curve of the variable elastic band changes based on the webbing length in each segment. Band A represents a single rubber band with length a, while Band B represents a band with length 2a. Bands C, D, and E represent variable elastic bands with two segments, each with a rubber band with length a. The lengths of the webbings for Bands C, D, and E are defined as follows:

(1) $$w_{C}=a$$

(2) $$w_{D}=a+\frac{l}{2}$$

(3) $$w_{E}=a+\frac{l_{1}}{2}$$

(4) $$0<l_{1},\;l_{2}<l$$

(5) $$l=l_{1}+l_{2}$$

Here, $w_{C}$, $w_{D}$, and $w_{E}$ represent the webbing lengths in the second segment of Bands C, D, and E, respectively, while l denotes the total elongation length, and $l_{1}$ and $l_{2}$ represent the initial and subsequent elongation lengths of Band E.

Unlike typical solid substances, rubber is a hyperelastic material with a nonlinear stress–strain curve, which is expressed using the Mooney–Rivlin model [14,18,19]:

(6) $$\sigma_{eng}=({2C}_{1}+{2C}_{2}\lambda^{-1})(\lambda \;-\;\lambda^{-2})$$

where $\sigma_{eng}$ is the engineering stress or the force F divided by the initial cross-sectional area $A_{0}$; ${2C}_{1}$ and ${2C}_{2}$ are material constants; and λ is the stretch ratio, equal to the strain ε plus one.

(7) $$F=A_{0}({2C}_{1}+\frac{{2C}_{2}}{\left(\varepsilon +1\right)})((\varepsilon +1)-\frac{1}{{\left(\varepsilon +1\right)}^{2}})$$

Therefore, for the same material and cross-sectional area, the force is identical if the strain is the same. As depicted in Figure 2, Band A stretches by l from an initial length of a, while Band B stretches by l from a length of 2a. As the corresponding strains are l/a and l/2a, respectively, Bands A and B have different force–elongation curves.

For Band C, the webbing length in the second segment matches the rubber band length; hence, only the first segment stretches by l. Consequently, the overall strain on Band C is l/a, and, thus, it exhibits the same force–elongation curve as Band A. For Band D, the webbing in the second segment is l/2 longer than the rubber band; this causes each segment to stretch by l/2, for a total extension of l across two segments. As this results in an overall strain of l/2a, Band D exhibits the same curve as Band B. For Band E, the webbing in the second segment is $l_{1}/2$ longer; therefore, each segment initially stretches by $l_{1}/2$, resulting in a total extension of $l_{1}$ and, consequently, the same curve as that of Band B. Once the length of the webbing in the second segment matches that of the rubber band, only the first segment is stretched by $l_{2}$ from $l_{1}$ to l; thereby, the curve resembles that of Band A.

This mechanism allows for adjusting the force–elongation curve to match that of either Band A or B, depending on the webbing length. By adjusting $l_{1}$, a variable curve transitioning from Band B to A can be generated, which provides multiple deformation possibilities.

The actual variable elastic band, shown in Figure 1, has one initial-length-adjustment segment and three elastic segments, and the webbing lengths can be adjusted using the three webbing control modules. This enables the implementation of four discrete sets of stiffness levels, based on Bands C and D in Figure 2, without any transition points. By combining this mechanism with the three transition points determined by the webbing lengths, numerous stiffness levels can be generated. More detailed explanations are provided in the Experiments and Results section.

### 2.3. Setting the Segment Lengths of the Variable Elastic Band

To create the variable elastic band, both the structure and operating principle of the band, as well as the segment lengths, must be considered. The segment lengths of the band determine the magnitude of the discrete stiffness levels. To clearly distinguish the various combined stiffness levels, the intervals between the discrete stiffness levels must be uniformly spaced. However, since rubber is a hyperelastic material that does not exhibit a linear elastic force, it is difficult to determine the segment length theoretically. Therefore, after measuring the stiffness levels of the rubber band on a test bench, the strain values corresponding to the 1/3 and 2/3 force points were identified. Then, while keeping the elongation length constant, the initial length was adjusted to achieve the desired strains.

**Figure 3:**
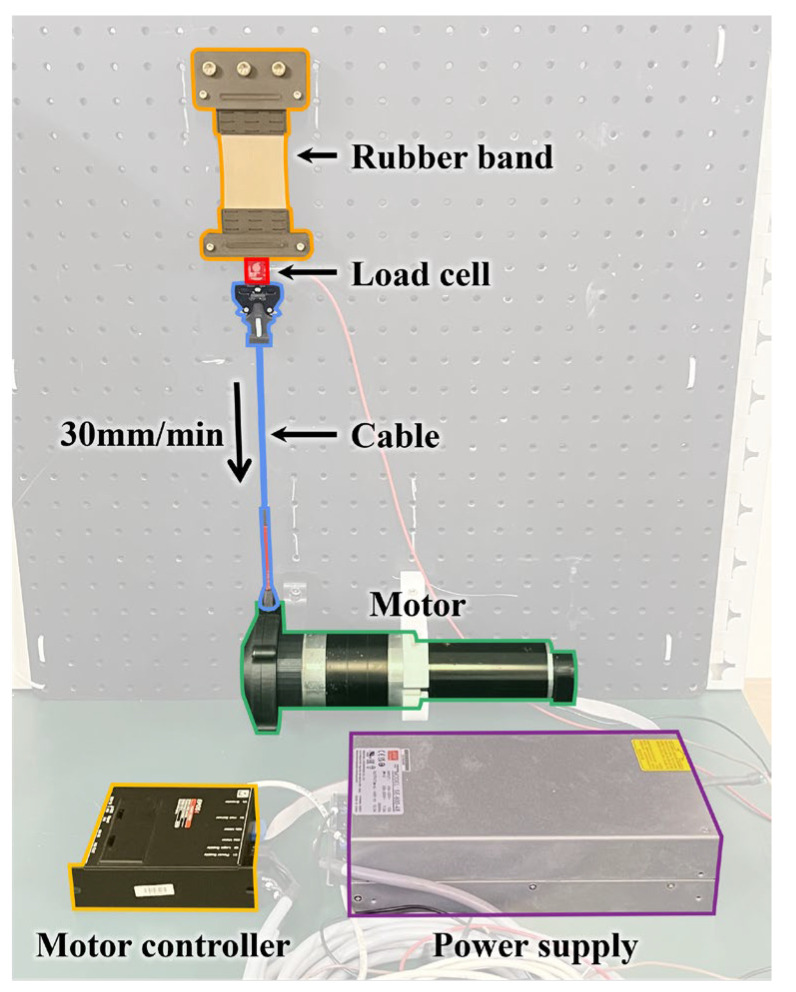
Test bench setup for force–elongation curve measurement.

The test bench setup for measuring the force–elongation curve is shown in Figure 3. The base plate was fixed vertically to prevent errors from sagging due to gravity. Additionally, to eliminate the effect of the rubber band’s weight on the load cell data, the upper end of the rubber band was fixed to the base plate, while the lower end was connected to a cable via a load cell (LSB205, Futek, Irvine, CA, USA). The elastic force was measured by pulling the cable using a motor (RE50, Maxon, Sachseln, Switzerland), while the load cell recorded the force. To capture the inherent properties of the rubber band accurately, it was stretched slowly at 30 mm/min. As the variable elastic band is modeled using the Mooney–Rivlin model, which has been reported to be valid up to 100% strain, the band was stretched to 100% strain [19]. As the Mooney–Rivlin model is not reliable beyond 100% strain, the elastic band was designed to operate strictly within this strain range, ensuring that all predicted forces remain within the valid domain of the material model.

**Figure 4:**
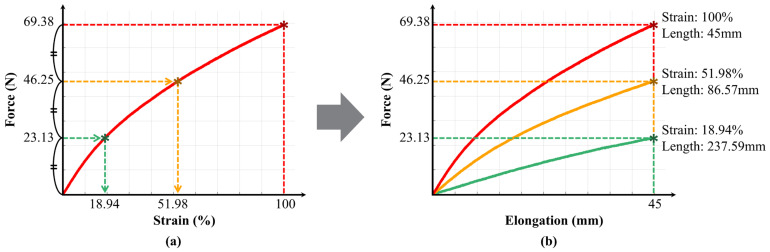
Selection of segment lengths for the variable elastic band. (**a**) Force–strain curve of the rubber band obtained from tensile testing. The strain values corresponding to 1/3 and 2/3 of the maximum force are indicated (x-axis: strain [%], y-axis: force [N]). (**b**) Selection of initial webbing lengths for generating equal intervals between discrete stiffness levels at a reference elongation length of 45 mm (x-axis: elongation [mm], y-axis: force [N]).

Figure 4a shows the measured force–elongation curve of the rubber band, with the *x*-axis representing strain (the ratio of elongation to the maximum elongation). The maximum force at 100% strain was 69.38 N. To ensure equal intervals between the discrete stiffness levels, the segment lengths were set to generate forces of 23.13 N and 46.25 N, corresponding to 1/3 and 2/3 of 69.38 N, respectively. These values correspond to the following strain levels: 100% strain for 69.38 N, 51.98% strain for 46.25 N, and 18.94% strain for 23.13 N.

The rubber band length that produces a 100% strain was determined based on the maximum elongation during negative work when the variable elastic band is worn, considering its application in a hip-flexion exosuit. The elongation length was calculated to be 45 mm based on data provided by J. Kim et al. [20], considering hip flexion and extension angles of approximately 10–16°.

The strain is the elongation length divided by the initial length. Since the elongation length was set to 45 mm, dividing the elongation length by the target strain yielded the corresponding initial length. The calculated initial lengths and corresponding force–elongation curves are shown in Figure 4b. The length of Segment 1, creating a strong stiffness, was 45 mm, calculated by dividing 45 by 1 to achieve 100% strain. The combined length of Segments 1 and 2, creating a moderate stiffness, was 86.57 mm, calculated by dividing 45 by the strain of 51.98%. The combined length of Segments 1, 2, and 3, creating a weak stiffness, was 237.59 mm, calculated by dividing 45 by the strain of 18.94%. Ultimately, Segments 1, 2, and 3 are 45 mm, 41.57 mm, and 151.02 mm, respectively.

## 3. Experiments and Results

### 3.1. Experimental Validation of Stiffness Levels of Variable Elastic Band

Before incorporating the variable elastic band into the hip-flexion exosuit, we conducted tests to measure and predict the varying stiffness levels of the band. The purpose of this experiment was to verify whether the variable elastic band could actually generate a spectrum of stiffness levels and to assess whether the numerous possible stiffness levels generated by different motor positions could be accurately predicted.

The experiment was conducted by fixing the variable elastic band to the test bench, as shown in Figure 3, and using a motor to stretch the band up to 45 mm at a speed of 30 mm/min. The elastic force was measured using a load cell during this process.

**Figure 5:**
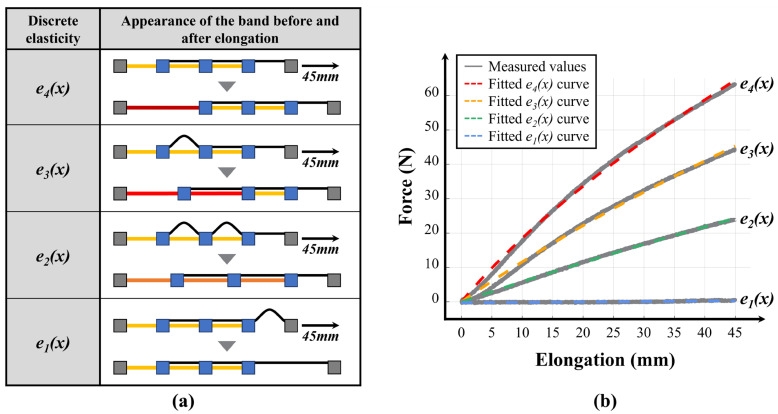
Formulation of the four single discrete elasticities of the variable elastic band. (**a**) Appearance of the band before and after elongation for each discrete stiffness level. The color of the band after elongation represents higher strain levels in the order of dark red, red, and orange. (**b**) Graph showing the experimentally measured values and the fitted Mooney–Rivlin model.

The variable stiffness levels can be predicted based on the four discrete stiffness levels that the band can generate, and the three stiffness transition points determined by the motor positions. To achieve this, tensile tests were first conducted with the webbing length settings that create the four discrete stiffness levels, as denoted by $e_{1}(x)$, $e_{2}(x)$, $e_{3}(x)$, and $e_{4}(x)$ in Figure 5a. The results are shown as gray solid lines in Figure 5b, which represent the four discrete stiffness levels measured. These measured stiffness levels were fitted to the Mooney–Rivlin model to derive the respective equations. Since the values obtained from the experiment are the forces corresponding to the elongation lengths in Equation (7), the strain was modified to be the elongation length x divided by the initial length $l_{0}$.

(8) $$F=A_{0}\left({2C}_{1}\left(\frac{x}{l_{0}}+1\right)-\frac{{2C}_{1}}{{\left(\frac{x}{l_{0}}+1\right)}^{2}}+C_{2}-\frac{C_{2}}{{\left(\frac{x}{l_{0}}+1\right)}^{3}}\;\right)$$

Here, $A_{0}$ is the cross-sectional area of the rubber band, which is 2 mm × 50 mm. According to the manufacturer’s datasheet, the thickness is 1/16″ (2.0 mm) with a tolerance of ±0.41 mm, and the width is 2″ (50.8 mm) with a tolerance of ±6.35 mm; $l_{0}$ is the initial length of the rubber band for each discrete stiffness level, specifically 45 mm, 86.57 mm, and 237.59 mm; x is the elongation length calculated from the motor encoder data; and F is the elastic force measured by the load cell. The detailed derivation from Equations (6)–(8) is provided in Supplementary Note S1. As seen in Figure 5a, the discrete stiffness level $e_{1}(x)$, which represents the outcome of adjusting the initial length without stiffness, could be ideally maintained at 0 N and was therefore fitted with a linear polynomial. $e_{1}(x)$, $e_{2}(x)$, $e_{3}(x)$, and $e_{4}(x)$ are shown as dashed lines in Figure 5b, and each was formulated using MATLAB’s Curve Fitting Toolbox (R2021b, MathWorks, Natick, MA, USA) as follows:

(9) $$e_{1}\left(x\right)=0.01246x\;-\;0.2679$$

(10) $$e_{2}\left(x\right)=100\left(0.6517\left(\frac{x}{237.59}+1\right)-\frac{0.6517}{{\left(\frac{x}{237.59}+1\right)}^{2}}-0.1763+\frac{0.1763}{{\left(\frac{x}{237.59}+1\right)}^{3}}\;\right)$$

(11) $$e_{3}\left(x\right)=100\left(0.5411\left(\frac{x}{86.57}+1\right)-\frac{0.5411}{{\left(\frac{x}{86.57}+1\right)}^{2}}-0.1884+\frac{0.1884}{{\left(\frac{x}{86.57}+1\right)}^{3}}\;\right)$$

(12) $$e_{4}\left(x\right)=100\left(0.4212\left(\frac{x}{45}+1\right)-\frac{0.4212}{{\left(\frac{x}{45}+1\right)}^{2}}-0.1055+\frac{0.1055}{{\left(\frac{x}{45}+1\right)}^{3}}\;\right)$$

The determination coefficients (R^2^) for each of $e_{1}(x)$, $e_{2}(x)$, $e_{3}(x)$, and $e_{4}(x)$ are 0.6664, 0.9991, 0.9962, and 0.9978, respectively, and their root mean square error (RMSE) values are 0.1143, 0.2146, 0.828, and 0.8685, respectively. At this point, the relatively low coefficient of determination for $e_{1}(x)$ is due to the very small variance of nearly constant force data in the form of a linear polynomial, and the RMSE of approximately 0.1 N indicates that the fitting was accurate.

**Figure 6:**
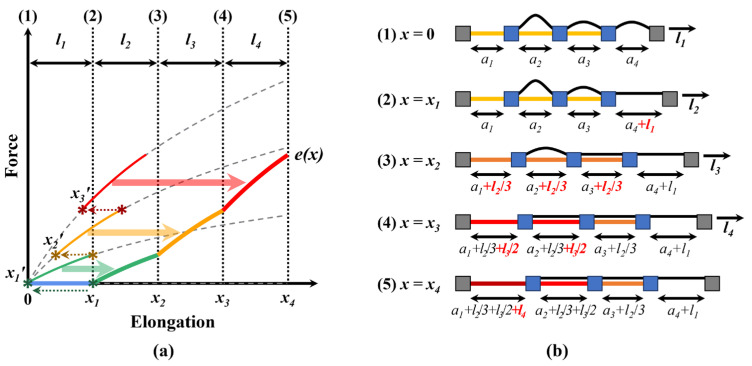
Method for predicting variable stiffness profiles based on the four discrete stiffness levels and three transition points. (**a**) Variable stiffness profiles predicted based on the discrete elasticities and transition points. (**b**) Step-by-step elongation of the variable elastic band.

Subsequently, as shown in Figure 6a, the variable stiffness function e(x) was predicted using the equations for $e_{1}(x)$, $e_{2}(x)$, $e_{3}(x)$, and $e_{4}(x)$, along with the transition points $x_{1}$, $x_{2}$, and $x_{3}$. From an elongation length of 0 to $x_{1}$, the variable stiffness follows the discrete stiffness $e_{1}(x)$. Then, at the elongation length $x_{1}$, it transitions to following the discrete stiffness $e_{2}(x)$. At this point, since the force must be continuous, the ${x_{1}}^{\prime }$ of $e_{2}(x)$, which corresponds to the same force as $e_{1}(x_{1})$, can be determined. Therefore, from an elongation length of $x_{1}$ to $x_{2}$, the variable stiffness follows the segment of $e_{2}(x)$ from ${x_{1}}^{\prime }$ to ${x_{1}}^{\prime }+(x_{2}-x_{1})$. Similarly, when the elongation length reaches $x_{2}$, the stiffness transitions to following the segment of $e_{3}(x)$ from ${x_{2}}^{\prime }$, which corresponds to the same force as $e_{2}({x_{1}}^{\prime }+(x_{2}-x_{1}))$, to ${x_{2}}^{\prime }+(x_{3}-x_{2})$. Finally, when the elongation length reaches $x_{3}$, the variable stiffness follows the segment of $e_{4}(x)$ from ${x_{3}}^{\prime }$, which corresponds to the same force as $e_{3}({x_{2}}^{\prime }+(x_{3}-x_{2}))$, to ${x_{3}}^{\prime }+(x_{4}-x_{3})$. Thereby, the entire variable stiffness profile can be predicted.

(13) $$e_{1}\left(x_{1}\right)=e_{2}\left({x_{1}}^{\prime }\right)$$

(14) $$e_{2}\left({x_{1}}^{\prime }+x_{2}-x_{1}\right)=e_{3}\left({x_{2}}^{\prime }\right)$$

(15) $$e_{3}\left({x_{2}}^{\prime }+x_{3}-x_{2}\right)=e_{4}\left({x_{3}}^{\prime }\right)$$

Based on these equations, the variable stiffness function e(x) can be predicted as follows:

(16) $$e\left(x\right)=\left\{\begin{array}{cc} e_{1}(x),\;\; & 0\leq x<x_{1}\\ e_{2}({x_{1}}^{\prime }+(x-x_{1})),\;\; & x_{1}\leq x<x_{2}\\ e_{3}({x_{2}}^{\prime }+(x-x_{2})),\;\; & x_{2}\leq x<x_{3}\\ e_{4}({x_{3}}^{\prime }+(x-x_{3})),\;\; & x_{3}\leq x\leq x_{4} \end{array}\right.$$

To compare the predicted values with the measured values, the variable stiffness profiles were tested on the test bench. For consistent verification, the transition points of the variable stiffness profiles were set to 11.25 mm, 22.5 mm, and 33.75 mm, which divide the maximum elongation length into four equal parts. The test was conducted on a total of 31 variable stiffness profiles, achieved by combining two, three, and four discrete stiffness levels, as shown in Table 2.

**Figure 7:**
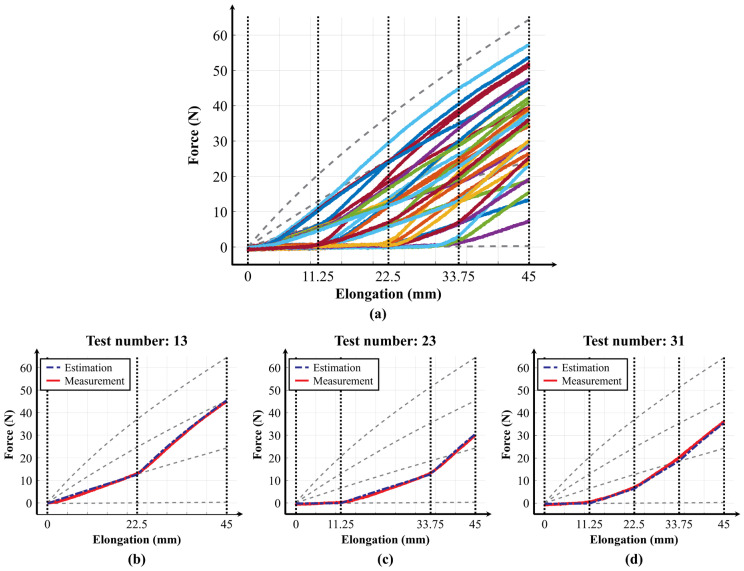
Experimentally measured stiffness profiles of the variable elastic band. (**a**) 31 variable stiffness profiles measured using the test bench (x-axis: elongation [mm], y-axis: force [N]). (**b**) Comparison of predicted and measured stiffness profiles combining two discrete stiffness levels. (**c**) Comparison of three discrete stiffness levels. (**d**) Comparison for four discrete stiffness levels.

Figure 7a shows the graphs of the measured values for the 31 variable stiffness profiles. Figure 7b, Figure 7c, and Figure 7d present representative graphs comparing the predicted and measured values of the variable stiffness profiles achieved by combining two, three, and four discrete stiffness levels, respectively. The full set of 31 comparison graphs is included in Supplementary Figure S1. Based on an analysis of these 31 stiffness profiles, the average coefficient of determination between the predicted and measured values was 0.9984 ± 0.0029 (mean ± standard error of the mean (s.e.m.)), and the average RMSE was 0.8993 ± 0.0911 (mean ± s.e.m.). These results confirm that the variable elastic band can generate a spectrum of elasticities and that these elasticities can be accurately predicted using the discrete stiffness equations and transition points.

### 3.2. Application of Variable Elastic Band in Hip Flexion Exosuit

**Figure 8:**
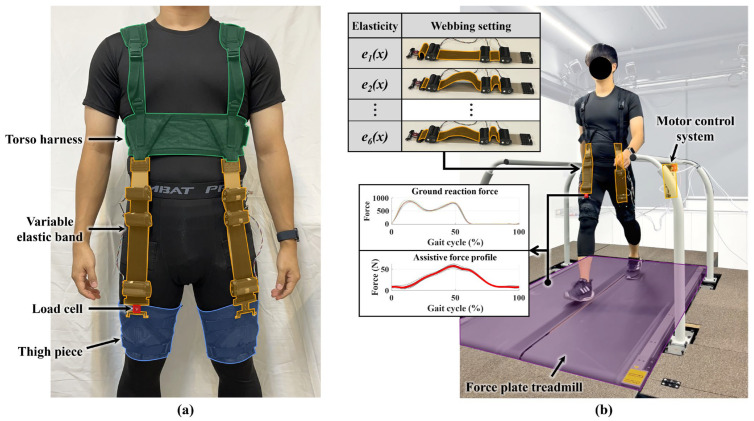
Experimental setup for measuring the assistive-force profile of the exosuit. (**a**) Components of the exosuit. (**b**) Measurement of the assistive-force profile of the exosuit. The webbing lengths are adjusted to create six different stiffness conditions, and the assistive-force profile is measured for each gait cycle using a force plate treadmill and load cell.

In this section, we discuss the testing of an exosuit with a variable elastic band, i.e., a hip-flexion exosuit incorporating the proposed variable elastic bands. As shown in Figure 8, the exosuit consists of torso harness and two thigh pieces. The bands were anchored by inserting the connectors into the lower attachment point of the torso harness and the upper attachment point of the thigh pieces, aligning them along the anterior midline of the thigh so that hip extension during walking produced band elongation and a corresponding flexion torque across the hip joint. The variable elastic bands are attached at both ends to the torso harness and thigh pieces, and the total mass of the exosuit is 999 g.

The objective of the experiment was to verify how the varying stiffness levels of the variable elastic band, which were validated in the test bench, influenced the assistive force profile when the device is worn. The experiment involved five healthy male participants (age: 26.8 ± 2.17 y, height: 1.69 ± 0.05 m, weight: 66.4 ± 4.51 kg; mean ± standard deviation). Extended demographic information is provided in Supplementary Table S1. Walking on a force plate treadmill at a speed of 1.5 m/s, the assistive force profiles generated by the variable elastic band were measured using load cells attached to the band. Prior to donning, the band-mounted load cells were zeroed under no-load conditions. Load cell and force plate signals were filtered using a fourth-order Butterworth low-pass filter with a 10 Hz cutoff implemented in MATLAB, and all gait cycles were normalized to 0–100% of the gait cycle based on heel strike detected from the force plate treadmill. Each participant walked 20 steps for each stiffness condition, and the gait cycle was segmented using data from the force plate. The assistive force profile was calculated by averaging the load cell data from the previous 10 steps. This study protocol was approved by the Institutional Review Board at Chung-Ang University (approval number 1041078-202107-HR-214-0, date of approval: 2 January 2025).

**Figure 9:**
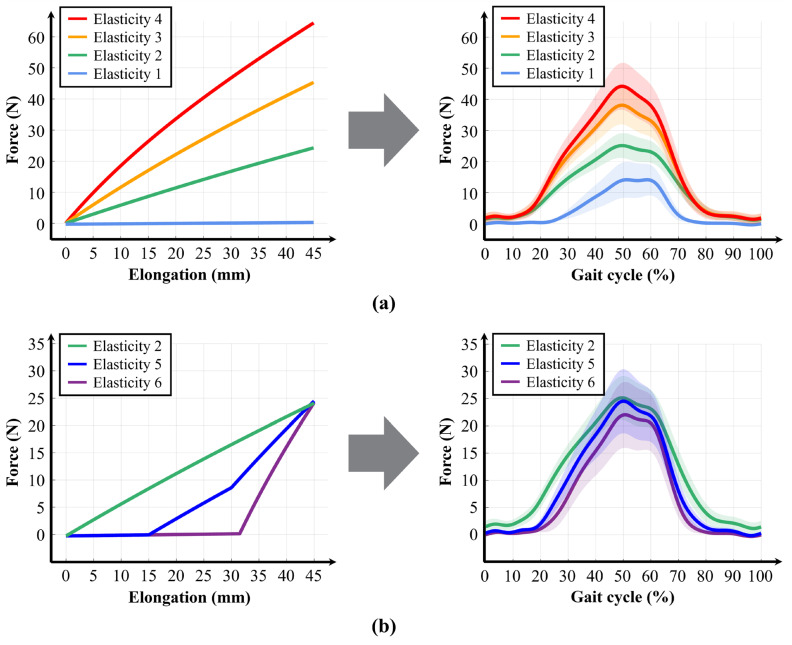
Experimental results of the hip-flexion exosuit equipped with the variable elastic band. (**a**) Changes in the magnitude of elastic force. Left: predicted values of input stiffness levels; Right: measured values from wearing experiments (x-axis: gait cycle [%], y-axis: force [N]). (**b**) Changes in the assistive force profile. Left: predicted stiffness profiles; Right: measured assistive force profiles obtained from five participants (x-axis: gait cycle [%], y-axis: force [N]). Shaded band regions represent the mean profile with surrounding widths corresponding to ± s.e.m. across participants.

The experiment was conducted under two conditions involving different stiffness levels: In the first experiment, the four discrete stiffness levels were compared to observe how variations in the stiffness magnitude affected the assistive force profile (Figure 9a). In the second experiment, three stiffness levels with similar maximum forces but different elastic profiles were selected to observe the impact of the elastic curve shape on the assistive force profile (Figure 9b).

Based on the results of the two experiments, the measured assistive force profiles exhibited trends similar to those observed in the test bench measurements. In the first experiment, the assistive force profile increased proportionally with the magnitude of the stiffness level, while, in the second experiment, the assistive force profiles similarly reflected the different stiffness profiles having the same maximum force but different shapes. These findings confirm that the variable elastic band enables various forms of passive assistance.

## 4. Discussion

This study demonstrated the variable elastic band mechanism’s ability to generate diverse elastic profiles and its potential to control the assistive force profiles of semi-passive exosuits. Through its compact design, a single variable elastic band unit weighed approximately 270 g, which is significantly lighter than the passive ankle exoskeleton [9], which ranges from 408 g to 503 g per side. The total mass of the exosuit, excluding tethered hardware, was 999 g. This mass difference of less than 400 g, compared to passive hip exosuits [6,7,8] with masses typically between 609 g and 645 g that assist hip flexion with rubber bands, enables the significant advantage of automatically adjusting a much wider range of elastic profiles. The variable elastic band operates at a very low force, less than 3.35% of the rubber tensile strength provided by the manufacturer. The webbing operates at a low speed of less than one revolution per minute and is aligned parallel to the band. Therefore, even with repeated use, no significant permanent deformation of the rubber, webbing bias, or friction-related issues were observed in bench tests or human wear tests. At the highest stiffness level, repeated cyclic tensile tests also showed consistent maximum forces across cycles, indicating stable viscoelastic behavior and confirming that no significant degradation occurred during repeated use.

In terms of ergonomics, participants reported no discomfort or skin irritation during the trials. Pressure was mainly concentrated on the waist belt and thigh pieces, consistent with typical soft exosuit designs. Because the total band length is about 370 mm, subjects shorter than ~160 cm may have insufficient waist-to-thigh spacing for proper fit, suggesting the need for a more compact design in future work.

Furthermore, the proposed system provides diverse elastic profiles with substantially lower power consumption than fully active systems. According to the motor datasheet, the no-load current at 6 V is 0.15 A, and the maximum duration required for a single webbing adjustment is 1.818 s. Therefore, for three motors driving the variable elastic band, the energy consumption per adjustment is approximately 4.92 J. Since power is consumed only during profile adjustments and the system otherwise operates passively, it can function for extended periods with minimal energy consumption. Furthermore, during walking, the required torque remained below the motor’s maximum capability, allowing the webbing control module to adjust the length without noticeable instability.

As shown in Table 3, this system overcomes not only the limitations of previously introduced passive systems [6,7,8,9,10], which allow only manual adjustment of elastic magnitude but also the shortcomings of systems that automatically adjust only the magnitude [14,15] or allow customization of target force curves [16,17]. The experiment results confirmed a very high correlation between the stiffness profiles measured on the test bench and the predicted values, which indicates that the variable elastic band can accurately generate consistent elasticities.

Moreover, formal statistical analysis of the on-body experiment (*n* = 5) confirmed that the assistive force increased systematically with stiffness level. A repeated measures ANOVA revealed a highly significant main effect of stiffness on the applied assistive force (F(3,12) = 50.21, *p* < 0.001, partial η^2^ = 0.93). Bonferroni-corrected pairwise comparisons further verified significant differences between all stiffness conditions, demonstrating that the variable elastic band provides systematically distinguishable levels of assistance during actual wear.

However, discrepancies between the applied stiffness levels and the measured assistive force profiles were observed in the variable-elastic-suit experiments. As shown in Figure 9a, while the applied maximum elastic forces were approximately 0 N, 24 N, 45 N, and 64 N, the actual assistive forces were around 13 N, 25 N, 38 N, and 44 N. Excluding the reference condition, the measured force corresponded to approximately 104.17%, 84.44%, and 68.75% of the benchtop values for the three stiffness levels, respectively. The pattern in which the actual assistive force decreases relative to the expected value as the target force increases can partly be attributed to frictional and other energy losses, but a more significant factor is the shorter-than-expected elongation of the band caused by deformation at the clothing fixation site. Preliminary testing indicated that the flexibility of the skin and clothing under assistive loading induces temporary or permanent deformation, which progressively reduces the effective tensile length as the assistive force increases.

**Figure 10:**
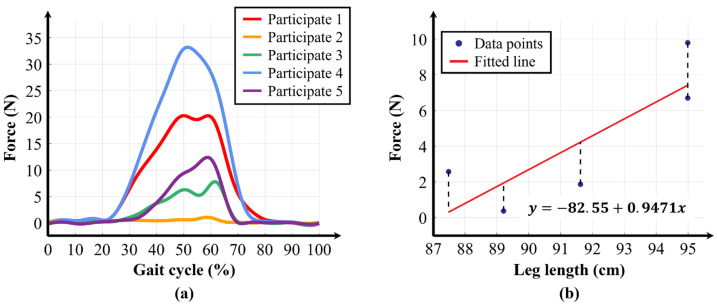
Analysis of assistive-force profile differences between participants for the same stiffness levels. (**a**) Assistive-force profiles for each participant when applying Elasticity 1. (**b**) Linear regression analysis graph to evaluate the correlation between participants’ leg length and the average assistive force of Elasticity 1.

Additionally, as seen in Figure 10a, even when the same stiffness levels were applied, the assistive force profiles varied depending on the participant. This result indicates that the variable elastic band was stretched either more or less than the expected 45 mm for each participant. The elongation length is closely related to the individual’s physical characteristics, particularly leg length. Although the same exosuit-wearing protocol was applied to all participants, anthropometric differences, particularly in thigh length, led to variations in fixation location along the thigh. Taller participants tended to secure the thigh piece more proximally, whereas shorter participants wore it closer to the knee. As a result, the effective path of the band during walking was altered, causing differences in elongation and assistive force.

To analyze the relationship between assistive force and leg length, we performed a linear regression analysis using the average assistance from each experiment and the participants’ leg lengths. Figure 10b shows the result of the linear regression analysis between elasticity 1 and leg length, which had the highest coefficient of determination (R^2^) of 0.6790. Table 4 presents the R^2^ values for each stiffness level, with an overall average of 0.5263. The information on the average assistance corresponding to each condition and leg length for each participant is provided in Supplementary Table S2. These results confirm that leg length has a statistically significant correlation with stiffness level. Additionally, when comparing the R^2^ values across different stiffness levels, a trend of decreasing R^2^ values was observed as the stiffness level increased. This suggests that, as stiffness level increases, other factors such as garment deformation, in addition to leg length, also influence the elongation length.

Despite the promising capabilities of the proposed system, there are inherent limitations in the current design. The variable elastic band mechanism is based on a serial configuration of rubber bands, where each segment contributes to constructing diverse elastic profiles. However, this serial arrangement inevitably increases the total length of the mechanism, which imposes constraints related to the wearer’s body size, particularly for users with shorter limbs or shorter torsos. Furthermore, the system’s efficiency is suboptimal, as it utilizes only a single segment of the rubber band to achieve its maximum force output. Currently, the assistive forces provided by the system are relatively modest. To generate greater forces, it is feasible to adjust both the cross-sectional thickness and tensile ratio of the rubber bands. Moreover, exploring more compact structural designs capable of producing diverse elastic profiles remains a promising area for future development.

Nevertheless, a significant advantage of this variable elastic band lies in its ability to adjust various elastic profiles with minimal power consumption. This feature opens new research opportunities: rather than manually tuning passive elastic forces in a limited and static manner, as done in previous studies, the variable elastic band can be leveraged to automatically explore a broad spectrum of assistive profiles. As such, more effective configurations may be discovered through systematic and real-time evaluation. This capability further introduces the possibility of applying HITL optimization strategies, previously restricted to active systems, in the passive domain. The proposed system enables real-time adjustment of assistance, allowing the search for optimal elastic profiles tailored to each user.

Moreover, passive exosuits have traditionally suffered from discrepancies between the predicted assistive force profiles from benchtop calibration and the actual profiles experienced during human use. These differences often arise from system deformation, anthropometric variability, and inconsistencies in how the device is worn. With the proposed variable elastic band, such mismatches can be mitigated by customizing the assistance profile post-wear, thereby achieving a closer match to the intended force trajectory.

Looking ahead, this technology may also be integrated with simulation-based approaches to identify optimal passive assistance strategies. Once an ideal passive torque or force profile is computationally determined, the variable elastic band can be configured to replicate and validate these profiles in real-world experiments. This bidirectional connection between simulation and physical implementation offers a powerful framework for advancing semi-passive exosuit development.

## 5. Conclusions

In this study, a novel variable elastic band mechanism capable of adjusting the elasticities of semi-passive exosuits was developed and validated. The mechanism controls the length of webbings connected in parallel with rubber bands to limit the extension length of the rubber bands. By connecting these segments in series, a wide range of elasticities can be generated.

Using a test bench, we measured and modeled the force–elongation curves corresponding to the discrete stiffness profiles generated by the variable elastic band. Subsequently, we set 31 variable stiffness profiles by combining four discrete curves with three transition points and compared the measured values with the predicted values derived from the discrete stiffness profiles and the transition points. The comparison showed an average coefficient of determination of 0.9984 and an average RMSE of 0.8993, confirming that the variable elastic band can generate elasticities with very high accuracy. Additionally, walking tests with a hip-flexion exosuit equipped with the variable elastic band verified the ability to generate appropriate ASSISTIVE forces by modulating the elasticities.

However, discrepancies between the applied stiffness levels and the actual assistive force profiles were observed, along with differences in assistive force profiles across participants for the same stiffness conditions. This suggests that physical characteristics such as leg length or garment deformation may influence the performance of the exosuit. This underscores the need for more precise adjustments to minimize these discrepancies and maximize the performance of the variable elastic suit. Rather than applying theoretically derived optimal stiffness profiles uniformly to all participants, the assistive force profile must be optimized on an individual basis. Future research will aim to enhance the system by developing a more compact design with a broader adjustment range, enabling greater adaptability to diverse body types. Additionally, personalized control strategies will be implemented to tailor assistive force profiles to individual users. Importantly, these studies will also incorporate functional human outcomes such as metabolic cost, hip flexor EMG, and gait biomechanics, in order to rigorously validate the practical benefits of the proposed system. Furthermore, by leveraging the system’s low-power, real-time tunability, simulation-based validation [21,22] and human-in-the-loop optimization [11,12,13], previously limited to active systems, will be extended to the domain of passive systems.

## Figures and Tables

**Table 1:** Bill of materials, mass distribution, and key specifications of the variable elastic band.

Subassembly	Components	Mass (g)	Notes / Role
Motors	3× Micro gearmotor, 6 V, 986.41:1	31.5	10.5 g each, with extended shaft
Encoders	3× Magnetic encoder, 12 CPR	4.5	1.5 g each, feedback for position loop
Drivers	3× DRV8838 motor driver	1	0.3 g each, PWM motor control
Controller	Teensy 4.1 (ARM Cortex-M7, 600 MHz)	15	Position loop, PWM generation
Spools	Aluminum drum (16.5 mm)	45	Provides 45 mm webbing displacement per revolution
Gears	Spur gear (m = 0.5, 34T, S45C)	31	Torque transmission
Structure	Printed housings, connectors	79	Guides webbing, fixation
Sensors	Load cell (Futek LSB205) + amplifier	9	Force measurement
Misc.	Bands, webbings, bolts, bearings, etc.	54	-
Total	-	270	Verified mass of full assembly

**Table 2:** List of elastic transition points for the variable stiffness profile measurement experiment.

Number of Discrete Stiffness Levels Combined	Test Number	*x*_1_ (mm)	*x*_2_ (mm)	*x*_3_ (mm)	*x*_4_ (mm)
2	1	11.25	45	45	45
	2	11.25	11.25	45	45
	3	11.25	11.25	11.25	45
	4	22.5	45	45	45
	5	22.5	22.5	45	45
	6	22.5	22.5	22.5	45
	7	33.75	45	45	45
	8	33.75	33.75	45	45
	9	33.75	33.75	33.75	45
	10	0	11.25	45	45
	11	0	11.25	11.25	45
	12	0	22.5	45	45
	13	0	22.5	22.5	45
	14	0	33.75	45	45
	15	0	33.75	33.75	45
	16	0	0	11.25	45
	17	0	0	22.5	45
	18	0	0	33.75	45
3	19	11.25	22.5	45	45
	20	11.25	33.75	45	45
	21	22.5	33.75	45	45
	22	11.25	22.5	22.5	45
	23	11.25	33.75	33.75	45
	24	22.5	33.75	33.75	45
	25	11.25	11.25	22.5	45
	26	11.25	11.25	33.75	45
	27	22.5	22.5	33.75	45
	28	0	11.25	22.5	45
	29	0	11.25	33.75	45
	30	0	22.5	33.75	45
4	31	11.25	22.5	33.75	45

**Table 3:** Comparison of active, passive, and stiffness-modulated systems with the proposed variable elastic band.

System Type	Active System	Passive System [6,7,8,9,10]	Variable Stiffness System [14,15]	Custom Stiffness System [16,17]	Variable Elastic Band
Usage Duration	Limited	Unlimited	Long Duration Possible	Unlimited	Long Duration Possible
Elastic scale change	Yes	Yes	Yes	Yes	Yes
Curve shape change	Yes	No	No	Yes	Yes
Automatic adjustment	Yes	No	Yes	No	Yes

**Table 4:** The coefficient of determination for the linear regression analysis of leg length by stiffness level.

Stiffness Level	Coefficient of Determination (R^2^)
Elasticity 1	0.6790
Elasticity 2	0.5992
Elasticity 3	0.5250
Elasticity 4	0.4328
Elasticity 5	0.4667
Elasticity 6	0.4555
Average	0.5263

## Data Availability

The data are contained within the article and Supplementary Materials.

## Supplementary Materials

The following supporting information can be downloaded at: https://www.mdpi.com/article/10.3390/biomimetics10110734/s1, Note S1: Derivation from Equations (6)–(8); Figure S1: Graphs comparing the predicted and measured values of the 31 variable elastic properties; Table S1. Extended demographic information of participants; Table S2: The average assistance and leg length of each participant; and Video S1: Development of Variable Elastic Band with Adjustable Elasticities for Semi-Passive Exosuits.
