# Supplementary material for: Development of Variable Elastic Band with Adjustable Elasticities for Semi-Passive Exosuits
Source: Biomimetics (Basel). 2025 Nov 1;10(11):734. doi: 10.3390/biomimetics10110734 (PMC12650684; doi:10.3390/biomimetics10110734)
Supplement: Supplementary file 1 [file biomimetics-10-00734-s001.zip › biomimetics_VEB_Supplementary Material_Clean.pdf]

Note S1. Derivation from Eq. (6) to Eq. (7) and Eq. (8)

We provide here the detailed derivation from Eq. (6) to Eq. (8), clarifying the relation between engineering stress, force, and strain ratio.

$$\sigma_{eng} = (2C_1 + 2C_2\lambda^{-1})(\lambda - \lambda^{-2})$$

where λ is the stretch ratio. The engineering stress is related to force and initial cross-sectional area by

$$\sigma_{eng} = \frac{F}{A_0}$$

Since

$$\lambda = \varepsilon + 1$$

where ε denotes engineering strain, we obtain

$$F = A_0(2C_1 + \frac{2C_2}{(\varepsilon + 1)})((\varepsilon + 1) - \frac{1}{(\varepsilon + 1)^2})$$

$$F = A_0(2C_1(\varepsilon + 1) - \frac{2C_1}{(\varepsilon + 1)^2} + 2C_2 - \frac{2C_2}{(\varepsilon + 1)^3})$$

Finally, by substituting

$$\varepsilon = \frac{x}{l_0}$$

where x is the elongation and l_0 the initial length, Eq. (8) provides the force–elongation relationship used in the manuscript.

$$F = A_0\left(2C_1\left(\frac{x}{l_0} + 1\right) - \frac{2C_1}{\left(\frac{x}{l_0} + 1\right)^2} + C_2 - \frac{C_2}{\left(\frac{x}{l_0} + 1\right)^3}\right)$$

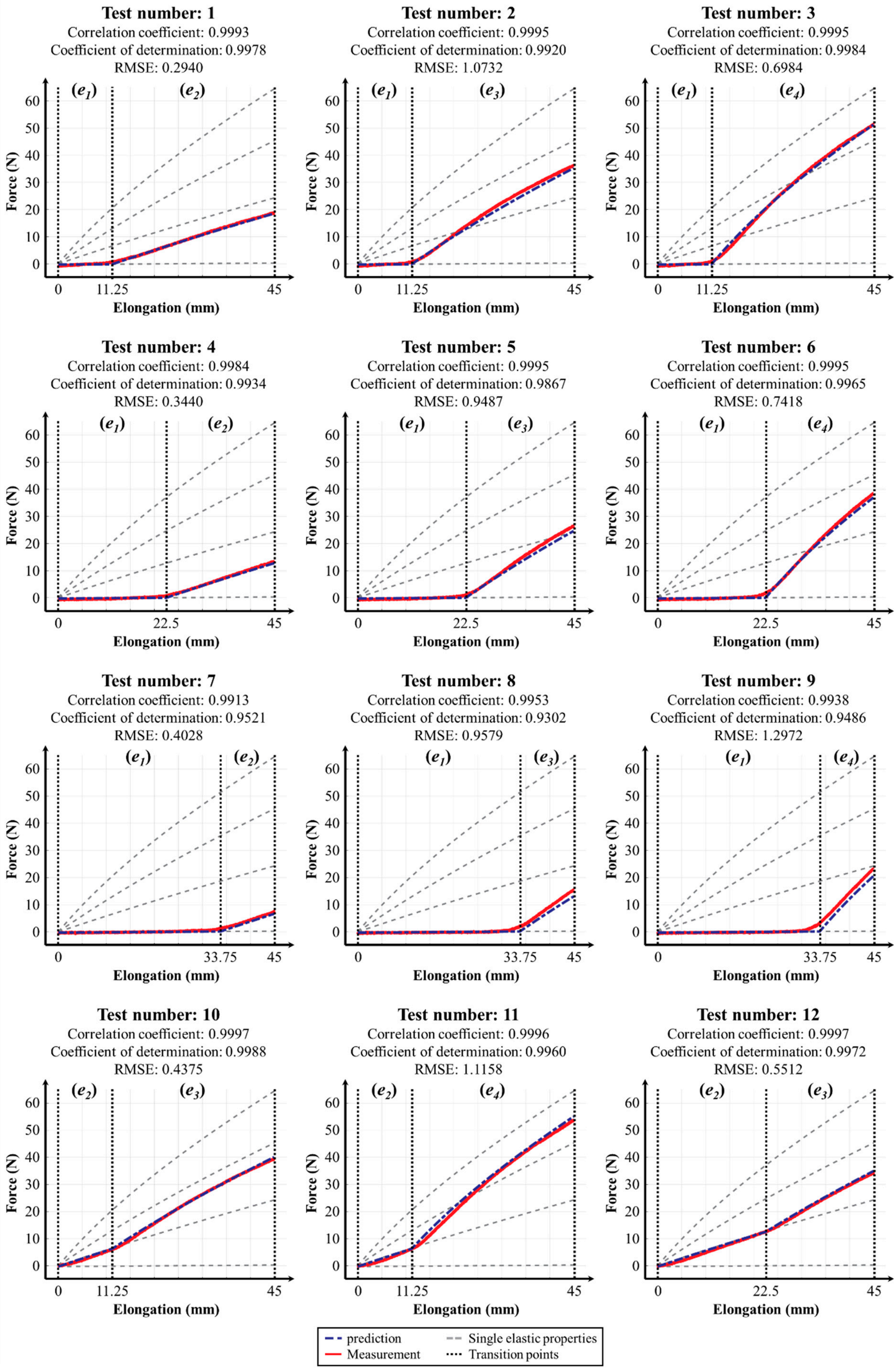

Test number: 1
Correlation coefficient: 0.9993
Coefficient of determination: 0.9978
RMSE: 0.2940
Test number: 2
Correlation coefficient: 0.9995
Coefficient of determination: 0.9920
RMSE: 1.0732
Test number: 3
Correlation coefficient: 0.9995
Coefficient of determination: 0.9984
RMSE: 0.6984
Test number: 4
Correlation coefficient: 0.9984
Coefficient of determination: 0.9934
RMSE: 0.3440
Test number: 5
Correlation coefficient: 0.9995
Coefficient of determination: 0.9867
RMSE: 0.9487
Test number: 6
Correlation coefficient: 0.9995
Coefficient of determination: 0.9965
RMSE: 0.7418
Test number: 7
Correlation coefficient: 0.9913
Coefficient of determination: 0.9521
RMSE: 0.4028
Test number: 8
Correlation coefficient: 0.9953
Coefficient of determination: 0.9302
RMSE: 0.9579
Test number: 9
Correlation coefficient: 0.9938
Coefficient of determination: 0.9486
RMSE: 1.2972
Test number: 10
Correlation coefficient: 0.9997
Coefficient of determination: 0.9988
RMSE: 0.4375
Test number: 11
Correlation coefficient: 0.9996
Coefficient of determination: 0.9960
RMSE: 1.1158
Test number: 12
Correlation coefficient: 0.9997
Coefficient of determination: 0.9972
RMSE: 0.5512
Force (N)
Elongation (mm)
prediction
Measurement
Single elastic properties
Transition points

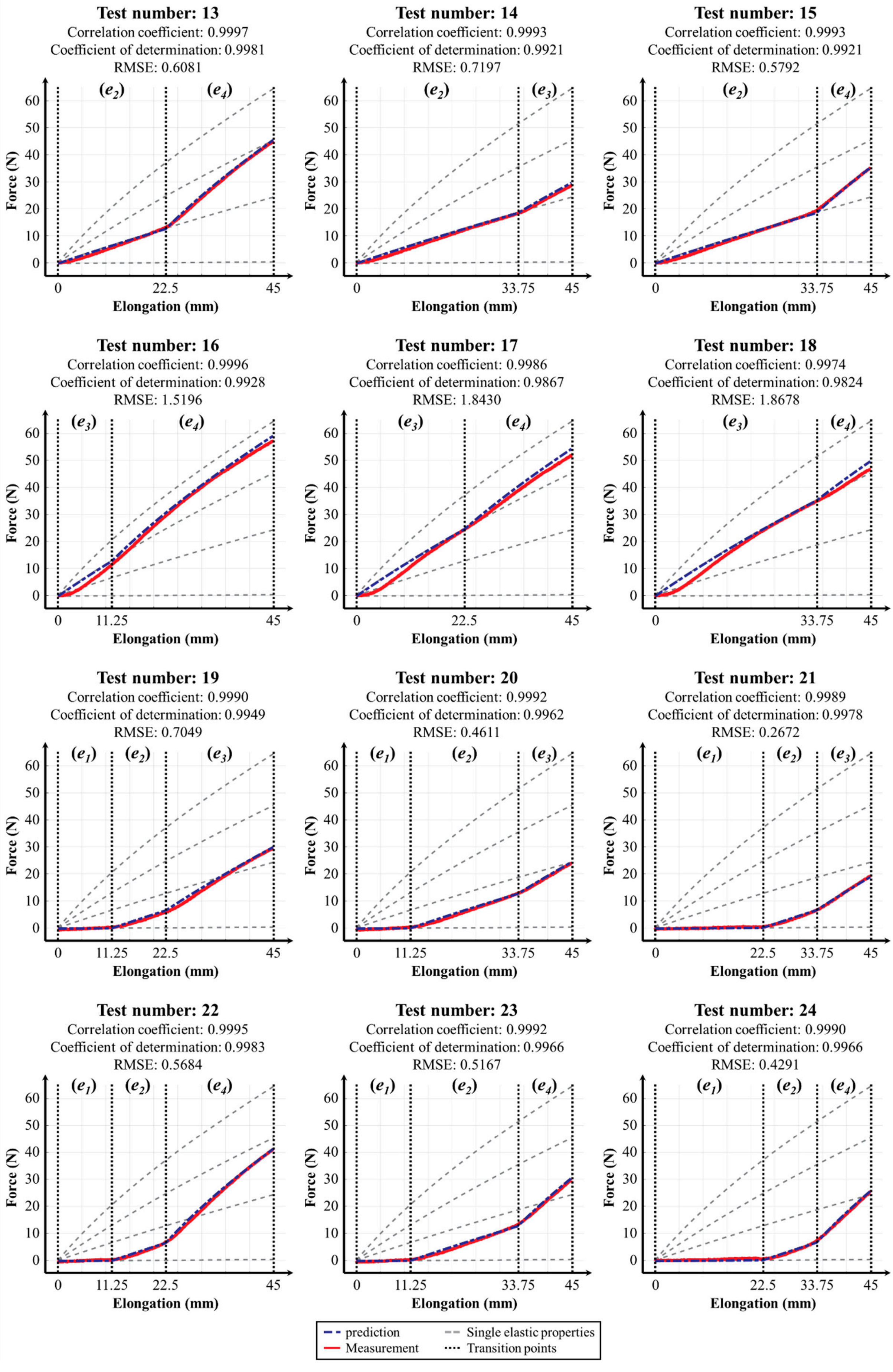
Test number: 13
Correlation coefficient: 0.9997
Coefficient of determination: 0.9981
RMSE: 0.6081
(e2) (e4)
Force (N)
Elongation (mm)
0 22.5 45
Test number: 14
Correlation coefficient: 0.9993
Coefficient of determination: 0.9921
RMSE: 0.7197
(e2) (e3)
Force (N)
Elongation (mm)
0 33.75 45
Test number: 15
Correlation coefficient: 0.9993
Coefficient of determination: 0.9921
RMSE: 0.5792
(e2) (e4)
Force (N)
Elongation (mm)
0 33.75 45
Test number: 16
Correlation coefficient: 0.9996
Coefficient of determination: 0.9928
RMSE: 1.5196
(e3) (e4)
Force (N)
Elongation (mm)
0 11.25 45
Test number: 17
Correlation coefficient: 0.9986
Coefficient of determination: 0.9867
RMSE: 1.8430
(e3) (e4)
Force (N)
Elongation (mm)
0 22.5 45
Test number: 18
Correlation coefficient: 0.9974
Coefficient of determination: 0.9824
RMSE: 1.8678
(e3) (e4)
Force (N)
Elongation (mm)
0 33.75 45
Test number: 19
Correlation coefficient: 0.9990
Coefficient of determination: 0.9949
RMSE: 0.7049
(e1) (e2) (e3)
Force (N)
Elongation (mm)
0 11.25 22.5 45
Test number: 20
Correlation coefficient: 0.9992
Coefficient of determination: 0.9962
RMSE: 0.4611
(e1) (e2) (e3)
Force (N)
Elongation (mm)
0 11.25 33.75 45
Test number: 21
Correlation coefficient: 0.9989
Coefficient of determination: 0.9978
RMSE: 0.2672
(e1) (e2) (e3)
Force (N)
Elongation (mm)
0 22.5 33.75 45
Test number: 22
Correlation coefficient: 0.9995
Coefficient of determination: 0.9983
RMSE: 0.5684
(e1) (e2) (e4)
Force (N)
Elongation (mm)
0 11.25 22.5 45
Test number: 23
Correlation coefficient: 0.9992
Coefficient of determination: 0.9966
RMSE: 0.5167
(e1) (e2) (e4)
Force (N)
Elongation (mm)
0 11.25 33.75 45
Test number: 24
Correlation coefficient: 0.9990
Coefficient of determination: 0.9966
RMSE: 0.4291
(e1) (e2) (e4)
Force (N)
Elongation (mm)
0 22.5 33.75 45
0 10 20 30 40 50 60
prediction
Measurement
Single elastic properties
Transition points

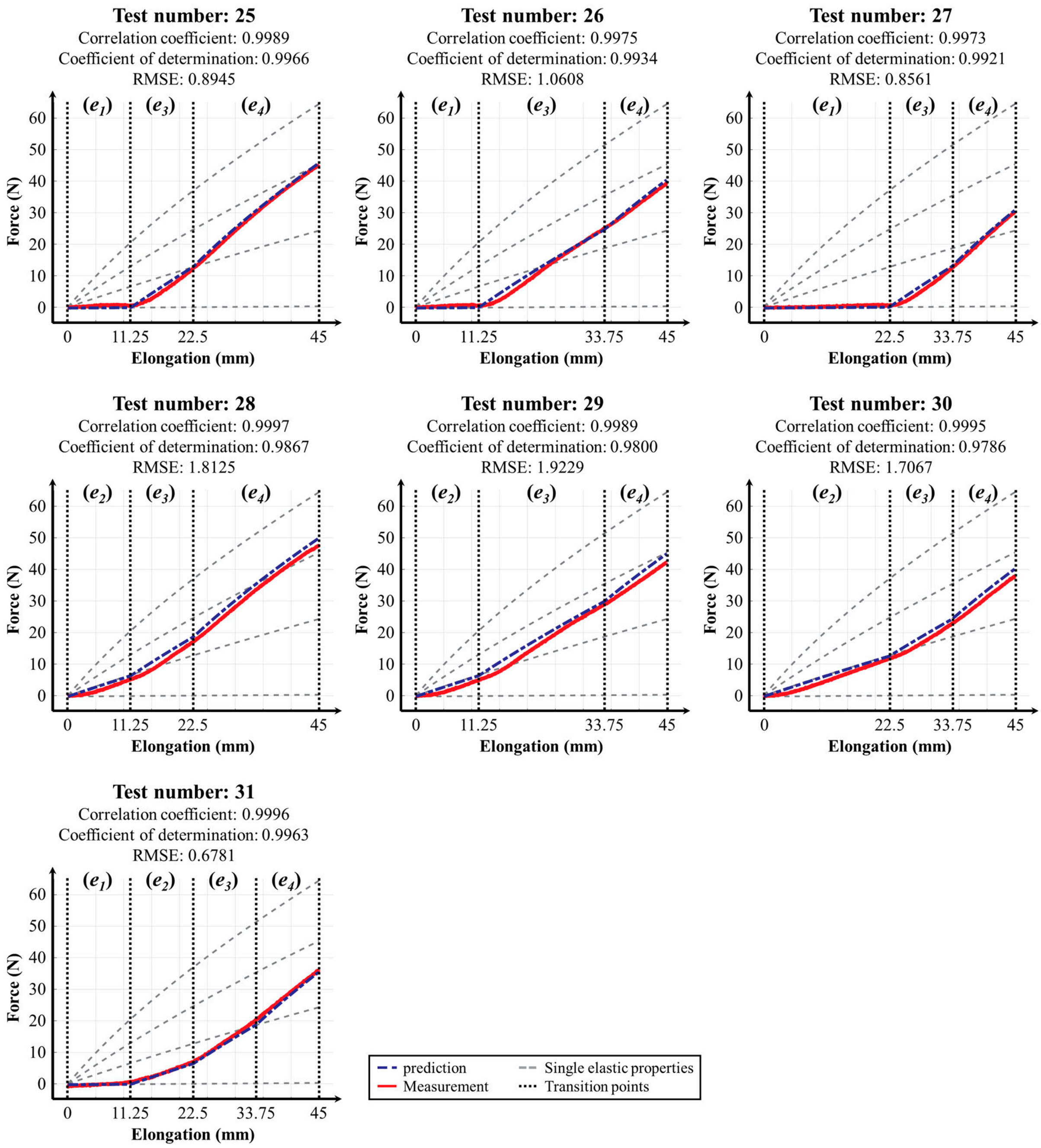

Figure S1. Graphs comparing the predicted and measured values of the 31 variable elastic properties. Each graph includes the test number, correlation coefficient, coefficient of determination, and RMSE. The blue dash-dotted line represents the predicted values, the red solid line represents the measured values, and the gray dashed lines represent the discrete elastic properties (e_1, e_2, e_3, and e_4). The black vertical dotted lines indicate the transition points for the test, with the corresponding discrete elastic property mentioned between the dotted lines.

Table S1. Extended demographic information of participants.

Subject	Sex	Age (years)	Height (cm)	Weight (kg)	BMI (kg/m^2)
Subject 1	Male	26	175	72	23.51
Subject 2	Male	29	166	64	23.23
Subject 3	Male	24	170	61	21.11
Subject 4	Male	26	175	65	21.22
Subject 5	Male	29	163	70	26.35

Table S2. The average assistance and leg length of each participant.

Subject	Elasticity 1 (N)	Elasticity 2 (N)	Elasticity 3 (N)	Elasticity 4 (N)	Elasticity 5 (N)	Elasticity 6 (N)	Leg length (cm)
Subject 1	6.6972	15.6493	23.3878	27.0557	12.7012	13.4492	94.988
Subject 2	0.3736	5.1746	6.861	6.7914	0.771	2.0241	89.209
Subject 3	1.8686	9.3697	11.9324	12.8484	4.8128	6.1504	91.623
Subject 4	9.7936	15.3117	21.1879	23.0331	10.1973	12.9764	94.988
Subject 5	2.5758	10.6152	15.6408	18.7175	7.5033	9.3521	87.486
